# (p)ppGpp-mediated stress response induced by defects in outer membrane biogenesis and ATP production promotes survival in ***Escherichia coli***

**DOI:** 10.1038/s41598-019-39371-3

**Published:** 2019-02-27

**Authors:** Mohammad Roghanian, Szabolcs Semsey, Anders Løbner-Olesen, Farshid Jalalvand

**Affiliations:** Centre for Bacterial Stress Response and Persistence, Department of Biology, University of Copenhagen, Ole Maaløes Vej 5, DK-2200 Copenhagen, Denmark

## Abstract

Cellular growth requires a high level of coordination to ensure that all processes run in concert. The role of the nucleotide alarmone (p)ppGpp has been extensively studied in response to external stresses, such as amino acid starvation, in *Escherichia coli*, but much less is known about the involvement of (p)ppGpp in response to perturbations in intracellular processes. We therefore employed CRISPRi to transcriptionally repress essential genes involved in 14 vital processes and investigated whether a (p)ppGpp-mediated response would be induced. We show that (p)ppGpp is produced and required for a pertinent stress response during interference with outer membrane biogenesis and ADP synthesis specifically. When these processes were perturbed via the transcriptional repression of essential genes, wild type *E. coli* MG1655 ceased growing and entered a semi-dormant state, whereas isogenic (p)ppGpp^0^ cells continued to grow uncontrollably to the point of lysis. Furthermore, *in vivo* measurements revealed that the ATP levels were intrinsically offset in (p)ppGpp^0^ cells, further indicating a role for the alarmone in cellular energy homeostasis. In summary, our investigation suggests that (p)ppGpp acts as a coordinator of cell growth in response to imbalances in outer membrane biogenesis and adenosine ribonucleotide synthesis, elucidating novel roles for (p)ppGpp in bacterial physiology.

## Introduction

Bacteria, as all other life forms, encounter a variety of stresses in nature that must be efficiently sensed and countered in order to ensure surviva^1^. One of the central stress responding pathways in *Escherichia coli* is the stringent response (SR) governed by the two homologous enzymes RelA and SpoT^2–4^. Both enzymes are synthetases of the alarmone nucleotides pppGpp and ppGpp (collectively referred to as (p)ppGpp), but only the bifunctional SpoT possesses (p)ppGpp-hydrolytic activity, rendering *spoT* conditionally essential in the presence of *relA*^2^. Upon production, the primary mode of action for (p)ppGpp is binding to the RNA polymerase to achieve a global re-wiring of the gene expression profile in response to stresses such as amino acid starvation^5^. In *E. coli* MG1655, (p)ppGpp induces a holistic transcriptional shift from genes encoding stable RNA and proteins directing macromolecular synthesis to those involved in general stress response pathways, *de novo* amino acid biosynthesis and re-assimilation of unused resources into the central metabolic pathways^5,6^. Overall, the accumulation of (p)ppGpp causes the differential expression of approximately 500 genes^6^, resulting in slow growth or dormancy.

The SR is also implicated in bacterial pathogenesis, host invasion, antibiotic tolerance as well as in responses to additional environmental stresses such as starvation for carbon sources, fatty acids, phosphate and during heat shock^2,7^. The alarmone has moreover been postulated to modulate and fine-tune general metabolism in bacteria during normal growth in the absence of external stresses, indicating involvement in a “checks-and-balances” type of growth regulation^2,8–10^. In investigated *Firmicutes*, namely *Enterococcus faecalis*, (p)ppGpp controls the pace of the carbon flow to govern the cellular response to external as well as internal metabolic cues^9,11^. But the role of (p)ppGpp in coordinating growth during intracellular process imbalances has not hitherto been well-characterized in the paradigmatic model organism *E. coli*.

Clustered regularly interspaced short palindromic repeats interference (CRISPRi) is a relatively novel molecular tool that allows for easy and efficient repression of gene transcription^12^. One of the major benefits of CRISPRi is the possibility of rapid transcriptional repression of virtually any gene without the need to introduce changes in the chromosome. In addition, the method is inducible, reversible and easily transferable to isogenic strains, rendering it a powerful instrument for dynamic studies of essential genes in bacteria.

**Figure 1:**
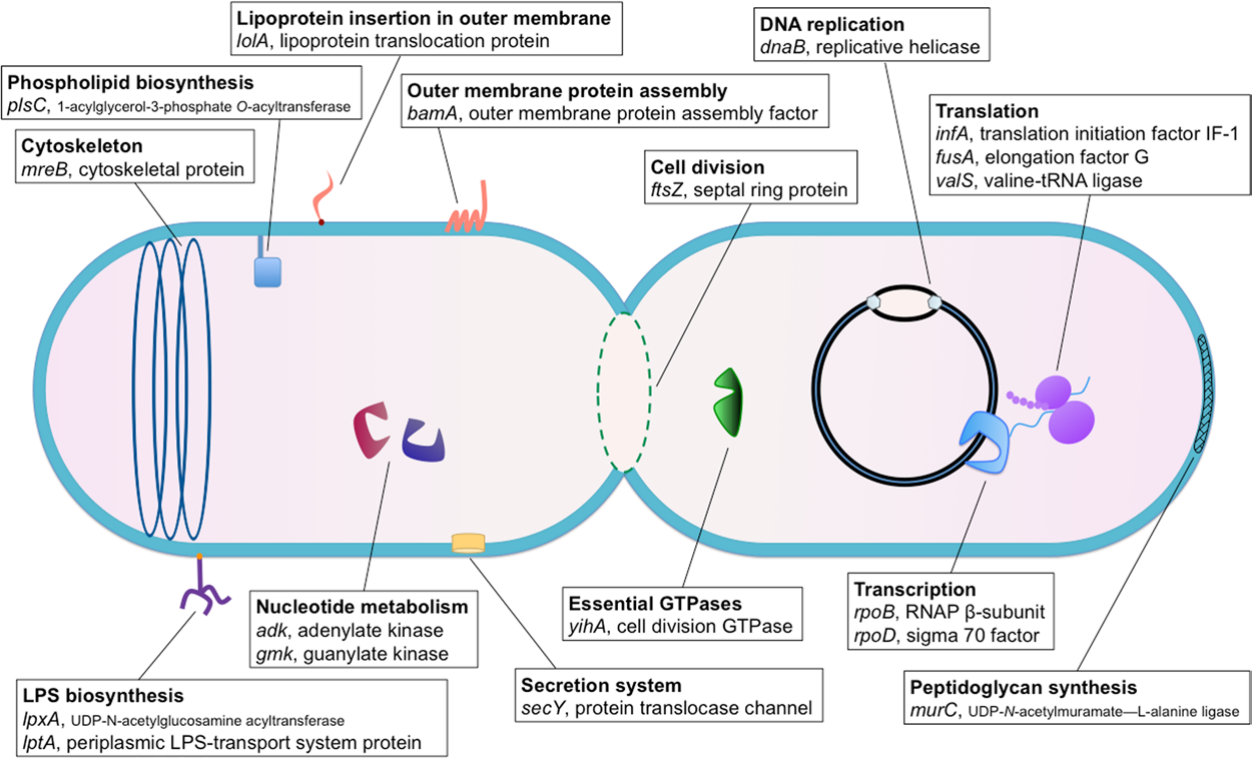
Schematic representation of the targeted cellular processes and genes. CRISPRi-mediated transcriptional repression of 18 essential genes involved in 14 specific cellular processes was carried out in MG1655. Activation of the stringent response was monitored during the induction of the specific intracellular imbalances.

In order to screen for novel roles for (p)ppGpp in cellular physiology, we employed CRISPRi to disrupt 14 essential cellular processes (cell division, cytoskeleton biogenesis, DNA replication, essential GTPase activity, lipoprotein incorporation in the outer membrane, lipopolysaccharides (LPS) biosynthesis and outer membrane biogenesis, nucleotide metabolism, outer membrane protein assembly, peptidoglycan synthesis, phospholipid biosynthesis, extracellular secretion, transcription, translation and tRNA aminoacylation) by repressing 18 essential genes in *E. coli* MG1655 (Fig. 1, Supplementary Table S1), collectively referred to as intracellular imbalances. We then performed single cell analyses, monitored SR activation via a fluorescent reporter and verified the synthesis of (p)ppGpp *in vivo* using thin-layered chromatography (TLC). We show that (p)ppGpp is produced and required for a pertinent stress response during disturbances in outer membrane biogenesis and ADP metabolism. Whereas wild type MG1655 (wt) enters a semi-dormant state during transcriptional repression of the pertinent genes, the isogenic Δ*relA*/*spoT* strain ((p)ppGpp^0^), incapable of producing the alarmone, grows in an uncontrolled manner, *i.e*. to the point of lysis when the same processes were perturbed. Collectively, our results implicate (p)ppGpp in regulation of cell growth during imbalances in outer membrane biogenesis and adenosine ribonucleotide synthesis in *E. coli*, indicating hitherto overlooked roles in bacterial physiology.

## Results

### Modification of the CRISPRi system

We hypothesized that the SR is involved in coordinating growth by transiently halting cellular proliferation when encountering various intracellular imbalances during active growth. To test our hypothesis, we produced CRISPRi constructs that via the transcriptional repression of 18 essential genes would introduce perturbations in 14 specific and vital cellular processes: cell division (*ftsZ*), cytoskeletal biogenesis (*mreB*), DNA replication (*dnaB*), essential GTPase activity (*yihA*), lipoprotein insertion in the outer membrane (*lolA*), outer membrane biogenesis (*lptA* and *lpxA*), nucleotide metabolism (*adk* and *gmk*), outer membrane protein assembly (*bamA*), peptidoglycan biosynthesis (*murC*), phospholipid biosynthesis (*plsC*), secretion (*secY*), transcription (*rpoB* and *rpoD*), and translation (*fusA* and *infA*) (Fig. 1). A detailed description of the genes, their encoded products, and the corresponding functions can be found in Supplementary Table S1. As a positive control for (p)ppGpp production we employed transcriptional repression of *valS*. Depletion of the valine-tRNA ligase ValS is a well-studied model for RelA synthetase activation via the ribosome-dependent uncharged tRNA-mediated mechanism^13–15^.

The CRISPRi method developed by Qi *et al*. is composed of a two-plasmid system comprising a gene-specific short guide RNA (sgRNA)-encoding plasmid (pgRNA) and an anhydrotetracycline (aTc)-inducible hydrolytically inactive Cas9 (dCas9)-encoding plasmid (pdCas9)^12^. When the sgRNA is co-expressed with *dcas9*, the products form a complex that efficiently blocks RNA polymerase from transcribing the gene targeted by the sgRNA. Upon applying the method to the essential genes of interest, we observed that the leakiness of P_Tet_ was producing sufficient amounts of dCas9 to kill the cells prior to the induction of the system. To circumvent the issue, a C-terminal ssrA-degradation tag^16^ was fused to *dcas9* to increase the turnover rate of the enzyme (Supplementary Fig. S1A,B). This approach eliminated the encountered problems associated with the background expression levels of *dcas9* as validated by the growth of all CRISPRi-containing strains during non-inducing conditions (Supplementary Fig. S1C). In parallel, when *dcas9* was induced, we observed the absence of growth, indicative of a proficient transcriptional repression of the essential genes in question. For the two genes *valS* and *yihA*, however, the repression did not abolish growth completely (Supplementary Fig. S1C). Finally, we included the non-essential *lacI* gene as a control and its repression did not result in any visible change in growth.

### Single cell analysis identifies *rpoS-mCherry* expression during disruption of ADP metabolism and outer membrane biogenesis

**Figure 2:**
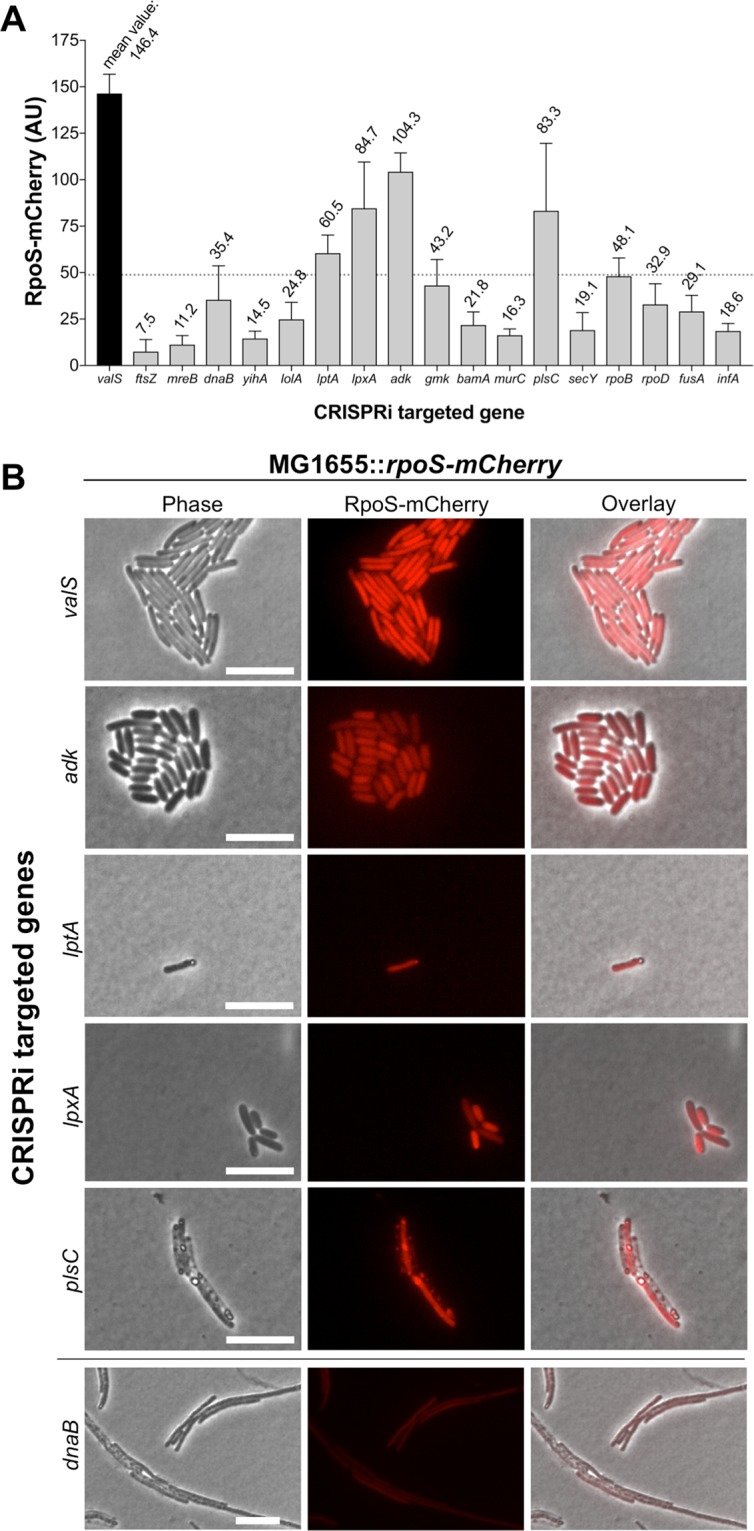
Single-cell analysis of RpoS-mCherry levels during the transcriptional repression of selected essential genes. CRISPRi-mediated shut down of expression of the indicated genes in a MG1655-derivate strain harboring the intracellular (p)ppGpp-reporter *rpoS-mCherry* (**A**) RpoS-mCherry fluorescence measurements for all investigated genes at the conclusion of the experiments at 15 h post-CRISPRi induction, or, when not applicable, just prior to the occurrence of lysis. Fluorescence was measured in >30 cells per experiment, the mean fluorescence intensities are shown. Error bars indicate standard deviations (SD). The pre-determined cutoff limit for hits we pursued is marked by the dotted line. (**B**) Phase contrast (left panels), the (p)ppGpp-reporter RpoS-mCherry fluorescence (middle panels) and the merged overlay images (right panels) capturing cells 15 h post-CRISPRi induction. The transcriptional repressions producing RpoS-mCherry levels exceeding the pre-determined cutoff limit are shown (*valS*; tRNA-charging, *adk*; nucleotide metabolism, *lptA* and *lpxA*; outer membrane biogenesis, and *plsC*; phospholipid biosynthesis). The phenotype of *dnaB* repression is shown as an example of the cellular process disturbances that did not yield a strong *rpoS-mCherry* expression for comparison. Scale bars = 10 µm.

Considering the intrinsic loss of viability that follows the repression of essential genes, we opted to screen single cells with time-lapse fluorescence microscopy to monitor the onset of the SR before death would occur. Therefore, CRISPRi constructs were introduced into a MG1655-derivative strain harboring a previously developed fluorescent (p)ppGpp-reporter consisting of the stationary phase sigma factor RpoS fused to mCherry^17^. The alarmone is a positive transcriptional regulator of *rpoS*^5^, rendering cellular RpoS levels an indirect indicator of cellular (p)ppGpp concentrations. Next, cells were brought to steady-state growth, spotted on solid M9 minimal medium agarose-pads supplemented with amino acids, glucose and *dcas9-*inducing aTc, and time-lapse microscopy was performed for up to 17 hours in temperature-controlled settings **(**Fig. 2A,B, Supplemental Fig. 2A–R**)**. Single cells were monitored for morphological changes, structural integrity and *rpoS-mCherry* expression.

CRISPRi-mediated disruptions of several well-characterized cellular processes resulted in predictable and previously reported phenotypes: *e. g*. filamentation in the case of cell division-^18^ and chromosomal replication inhibition^19^ (*ftsZ* and *dnaB*, respectively), stasis in the case of translational arrest^20^ (*fusA* and *infA)*, and the loss of rod shape in the case of cytoskeletal depletion^21^ (*mreB*) (Supplementary Fig. S2E–G, Q,R). Cell elongation was also observed during disruptions of lipoprotein insertion in outer membrane (*lolA*), GDP synthesis (*gmk*), β-barrel outer membrane protein assembly (*bamA*), and phospholipid biosynthesis (*plsC*) (Supplementary Fig. S2I–K, M). For the majority of the genes surveyed, various levels of lysis occurred within 15 hours (Supplementary Fig. S2). The fastest onset of lysis occurred during repression of *murC*, which caused the cells to lyse during the very first cell division event (presumably due to the lack of sufficient peptidoglycan monomers being synthesized) (Supplementary Fig. S2L). For the gene *yihA*, encoding an essential GTPase implicated in cell division^22^, no substantial effect of the transcriptional repression was detected as the cells grew uninhibitedly as also noted above. This suggested that the depletion of this GTPase was not substantial enough to generate a phenotype (Supplementary Fig. S2H).

As expected, the strongest *rpoS-mCherry* expression was observed during the repression of *valS* (mean fluorescence intensity (MFI) 146.4, ±10.4 standard deviation (SD)), indicative of (p)ppGpp accumulation (Fig. 2A,B, Supplementary Fig. S2A). The cellular growth rate was also significantly reduced in agreement with previous findings^14,23^, but not completely halted. We arbitrarily decided to pursue genes whose repression resulted in reduced growth rates and RpoS-mCherry values >1/3 of that observed during *valS* repression (Fig. 2A), corresponding to MFI > 48.8. Four genes met these criteria: *adk*, *lptA*, *lpxA* and *plsC* (Fig. 2, Supplementary Fig. S2B–D, M)

Transcriptional repression of *adk*, encoding adenylate kinase, the sole enzyme that catalyzes the conversion of AMP + ATP ↔ 2 ADP in the adenosine ribonucleotides *de novo* biosynthesis pathway^24^, resulted concomitantly in a notable reduction in cell size and a strong expression of *rpoS-mCherry* corresponding to MFI 104.3 (±10.1 SD) (Fig. 2B, Supplementary Fig. S2B). Similarly, CRISPRi-mediated repression of *lptA* and *lpxA*, encoding for key components in the biogenesis of the outer membrane, resulted in a strong expression of *rpoS-mCherry* corresponding to MFI 60.5 (±9.7 SD) and 84.7 (±24.8 SD), respectively (Fig. 2A,B, Supplementary Fig. S2C,D). Disruption of outer membrane biogenesis in an early- (*lpxA*) *vs* late stage (*lptA*) thus yielded similar phenotypes with regard to RpoS-mCherry production.

Consistent with an elevated (p)ppGpp level, greatly reduced growth rates or semidormancy was observed during repression of *adk*, *lptA* and *lpxA* (Supplementary Fig. S2B–D). The structural integrity of these cells was preserved throughout the duration of the experiment as evaluated by the retention of the cytoplasmic fluorescent reporter and the visual intensity of cells in phase-contrast images. However, although high levels of RpoS-mCherry were observed during transcriptional repression of *plsC* (MFI 83.3, ±37.2 SD), an enzyme that catalyzes the ligation of fatty acids into the 2-position of 1-acyl-glycerol-3-phosphate during phospholipid biosynthesis^25^, the gene was omitted from the downstream investigations due to the extensive cell lysis during its shut down (Supplementary Fig. S2M).

### (p)ppGpp is produced during transcriptional repression of *adk*, *lptA* and *lpxA*

It is known that the RpoS sigma factor is regulated on multiple stages, including post-transcriptionally and proteolytically, by factors independent of (p)ppGpp^26,27^. In light of these facts, the indications given by RpoS-mCherry as it pertains to intracellular (p)ppGpp levels need to be more directly corroborated.

**Figure 3:**
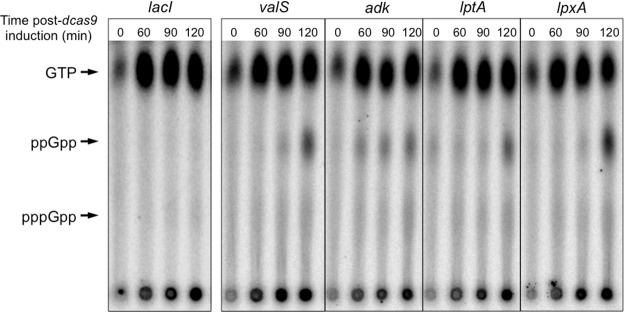
(p)ppGpp is produced *in vivo* during repression of *valS*, *adk*, *lptA* and *lpxA*. Autoradiographs of the lysate of radiolabeled cells showing (p)ppGpp synthesis over time during transcriptional repression of the indicated genes in MG1655. The experimental conditions were identical for all samples and they were processed in parallel. Representative examples of three independent experiments are shown.

To validate the findings of the time-lapse fluorescence microscopy, we employed thin-layered chromatography (TLC) to directly measure the *in vivo* (p)ppGpp levels in radiolabeled wt cells perturbed in the relevant cellular processes (Fig. 3). Repression of *lacI* and *valS* were employed as the negative and positive controls, respectively. As seen in Fig. 3, the SR was not activated during the repression of *lacI*, indicating that there is no intrinsic effect on alarmone production by the CRISPRi system. On the other hand, clear (p)ppGpp synthesis was observed during the repression of *adk*, *lptA* and *lpxA* genes, at levels comparable to *valS*. Repression of *adk* induced the fastest activation of the SR in this experimental set up, preceding the response elicited by *valS* repression. In summary, intracellular (p)ppGpp measurements substantiated the findings from the fluorescence microscopy indicating the activation of the SR during interferences with ADP metabolism and outer membrane biogenesis.

### (p)ppGpp is required for an efficient stress response during interference with LPS- and ADP synthesis

**Figure 4:**
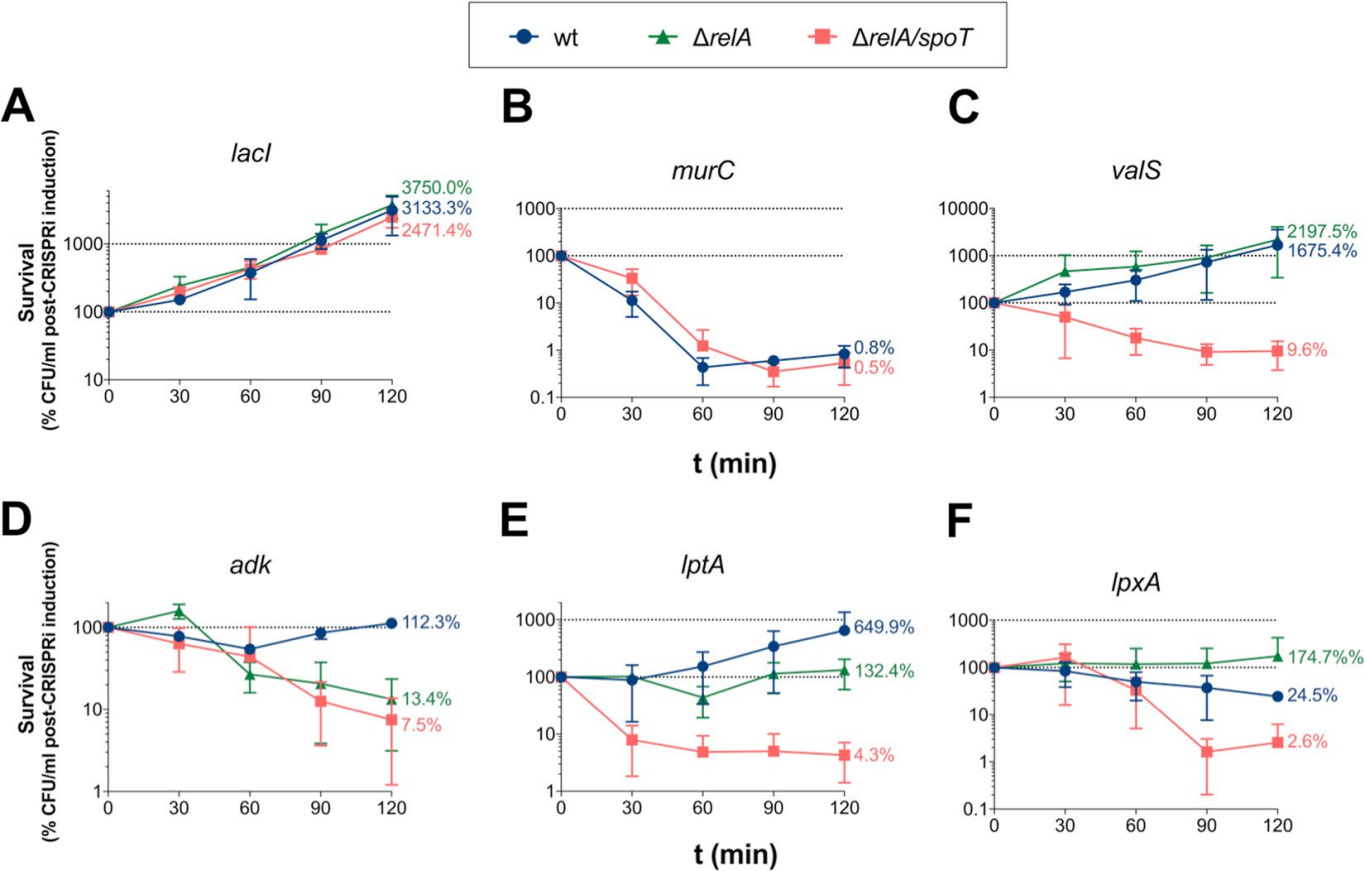
Cell viability during transcriptional repressions in MG1655 wt and isogenic background. Viable count of MG1655 wt (blue circles), Δ*relA* (green triangles) and Δ*relA/spoT* (red squares) isogenic mutants experiencing transcriptional repression of (**A**) *lacI*, (**B**) *murC*, (**C**) *valS*, (**D**) *adk*, (**E**) *lptA*, and (**F**) *lptX*. Values are given as the percentage of CFU/ml at the start of CRISPRi induction. The mean of three separate experiments is plotted, and error bars indicate the standard deviation. The endpoint means are stated in numbers.

The time-lapse microscopy indicated that cells experiencing repression of *valS*, *adk*, *lptA* and *lpxA* could remain viable in a reduced growth rate state for the duration of the experiment (Fig. 2). In order to determine whether the production of (p)ppGpp is a requisite for stress tolerance, a survival kinetics assay was performed in wt and isogenic Δ*relA* and Δ*relA/spoT* backgrounds (Fig. 4). The strains were brought to balanced growth in LB and *dcas9* was induced. Subsequently, samples were collected at the stated time points and plated on CRISPRi non-inducing media, allowing recovery from the transcriptional repression. Viable counts were performed the following day.

To ensure that the CRISPRi system was not intrinsically affecting cell viability in various isogenic backgrounds, CRISPRi of *lacI* was included in the assay as a control. *lacI*-repressed cells displayed normal growth kinetics for all three genotypes (Fig. 4A). Disruption of peptidoglycan monomer synthesis (via the repression of *murC*), conversely, revealed a rapid drop in the concentration of colony forming units (CFU) over time in both the wt and (p)ppGpp^0^ strain, indicating that there is no difference between the strains during this stress (Fig. 4B**)**. Hence, *murC* was not further evaluated in the Δ*relA* background.

However, major differences were seen in the isogenic backgrounds during CRISPRi of the remaining genes. First, repression of *valS* resulted in reduced growth rate (as compared to *lacI)*, in agreements with the observations from the time-lapse microscopy (Fig. 4C). A loss of viability was found when repressing *valS* in the Δ*relA/spoT* strain, expectedly demonstrating the necessity of (p)ppGpp for a proper stress response in circumstances mimicking amino acid starvation. A decrease in CFU/ml was, however, not observed in the isogenic Δ*relA*, which exhibited the same slow growth kinetics as wt, indicating that the presence of SpoT was sufficient to respond to the stress in this experimental set up.

Second, while cessation of growth was observed during interference with AMP → ADP conversion via the repression of *adk* in wt, both Δ*relA* and Δ*relA/spoT* failed to uphold viability while experiencing this stress. This suggests that (p)ppGpp synthesis is indeed required for sustained viability during ADP depletion, and in this circumstance, that the alarmone production is RelA-dependent (Fig. 4D).

Finally, depletion of LPS due to the repression of *lptA* and *lpxA* also resulted in reduced growth rates, in agreement with the data from the time-lapse microscopy (Fig. 4E,F). After 120 min of *lptA* repression, the mean viability was 650% for wt, 132% for Δ*relA*, but only 4% for the isogenic Δ*relA/spoT* (Fig. 4E). The corresponding endpoint mean values during *lpxA* repression were wt 25%, Δ*relA* 174%, and Δ*relA/spoT* 3% (Fig. 4F). Loss of viability was thus observed in the (p)ppGpp^0^ strain during repression of these genes, but not in the Δ*relA* background, indicating that the presence of SpoT was adequate to uphold cellular viability while experiencing perturbation in outer membrane biogenesis.

### Aberrant cell division in (p)ppGpp^0^ cells during repression of *valS*, *adk*, *lptA* and *lpxA*

**Figure 5:**
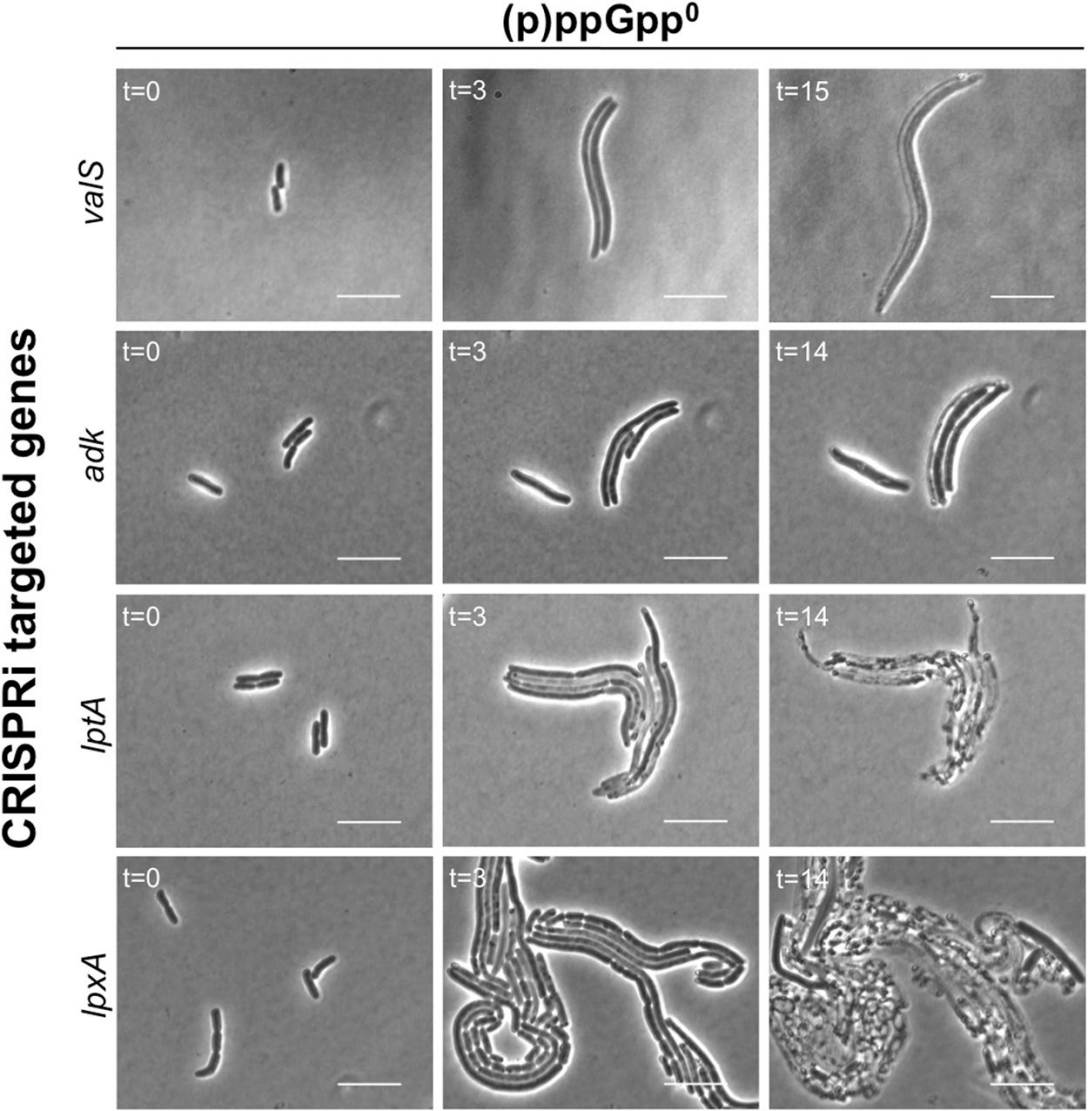
(p)ppGpp^0^ cells display aberrant growth and cell division during repression of *valS*, *adk*, *lptA* and *lpxA*. Time-lapse phase contrast imaging of Δ*relA/spoT* cells during transcriptional repression of the indicated genes. Scale bars = 10 µm.

To further explore the phenotype of the SR-deficient strain, time-lapse microscopy of was performed on the (p)ppGpp^0^ cells during repression of *valS*, *adk*, *lptA* and *lpxA*, *i.e*. in amino acid starvation-mimicking conditions, and during ADP- and LPS-depletion, respectively (Fig. 5). The strains were taken in balanced growth, spotted on LB agarose-pads supplemented with *dcas9*-inducing aTc, and time-lapse microscopy was performed for up to 15 h in temperature controlled-settings.

As seen in Fig. 5, various degrees of filamentation were observed within 3 h post-CRISPRi induction, indicating that cell division was inhibited but that cells continued to grow. This was in stark contrast to the phenotypes produced by (p)ppGpp^+^ cells, in which dormancy were induced, or where the rate of proliferation decelerated but cell division remained coordinated (Fig. 2, Supplementary Fig. S2A–D). Eventually, lysis was observed in all samples. In agreement with the previous results (Fig. 4), disruption of outer membrane biogenesis resulted in the most conspicuous loss of viability, as the cells exhibited rapid and filamentous growth until lysis would occur (Fig. 5).

**Figure 6:**
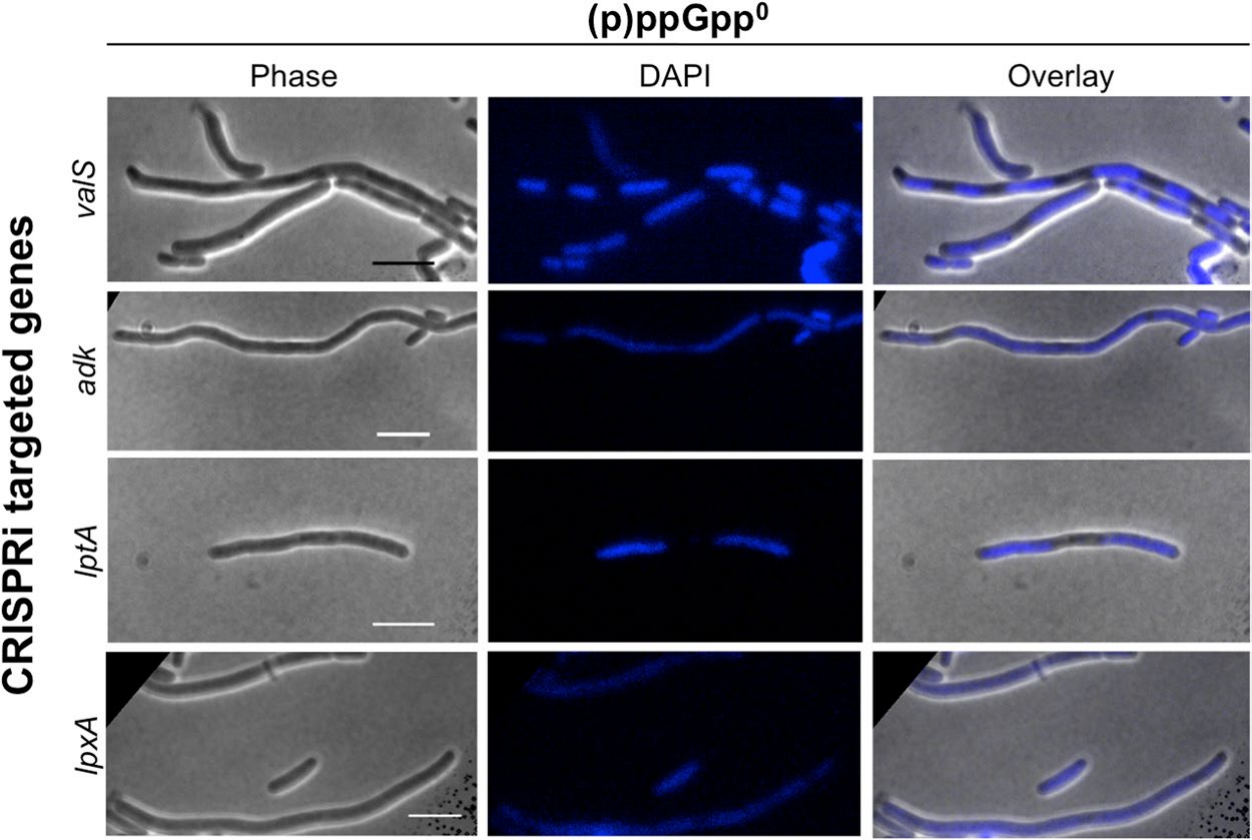
The nucleoid localizes differently in (p)ppGpp^0^ cells during repression of *valS*, *adk*, *lptA* and *lpxA*. Fluorescence microscopy imaging of DAPI-stained Δ*relA/spoT* cells 10 h post CRISPRi-mediated transcriptional repression of the indicated genes. Phase contrast (left panels), DAPI fluorescence (middle panels) and their merged overlay (right panels) are shown. Scale bars = 10 µm.

Next, we addressed whether there was a common cause of filamentation during shut down of the investigated pathways. It is known that the assembly of the Z ring is inhibited by the Min- and the nucleoid occlusions-systems; the latter involving the nucleoid-associated septal ring-assembly inhibitor SlmA that disallows Z rings to form in the vicinity of nucleoids^28^. To follow the localization of the DNA in the cell, CRISPRi was induced in (p)ppGpp^0^ cells and they were allowed to grow in supplemented liquid M9 minimal media at 37 °C without shaking (to not expose filamentous cells to undue force). At the indicated time points, samples were taken, nucleoids stained with DAPI, and fluorescence microscopy was performed (Fig. 6, Supplementary Fig. S3).

In a somewhat surprising finding, multiple replicated and fully segregated chromosomes could be seen in the filamentous cells experiencing a spike in uncharged tRNAs (*valS* repression), whereas the opposite was true for those perturbed in adenosine ribonucleotide synthesis (*adk*) and outer membrane biogenesis (*lptA* and *lpxA*), in which DNA were found more evenly spread throughout the elongated cells. These phenotypes were observed at 3 h (Supplementary Fig. S3) as well as 10 h post-CRISPRi induction (Fig. 6). The data indicate that no single mechanism underlies the filamentous phenotypes observed during CRISPRi repression of the studied genes.

### The intracellular ATP concentrations are perturbed in (p)ppGpp^0^ strain

**Figure 7:**
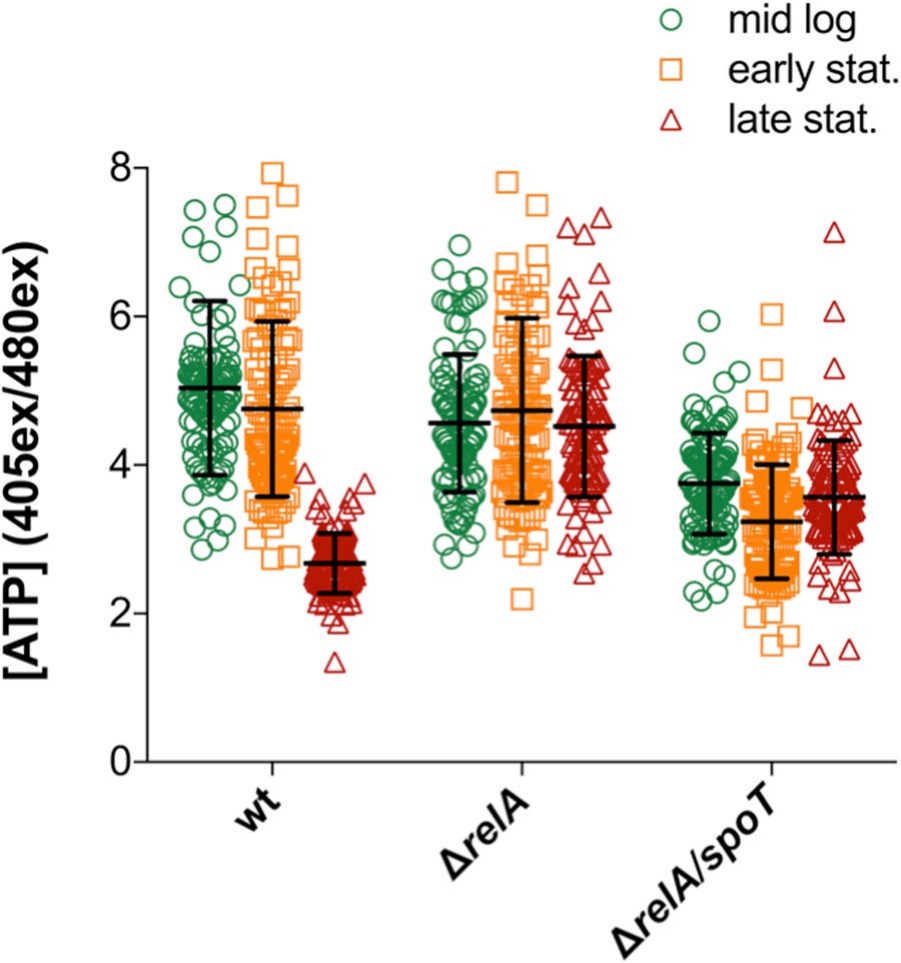
The intracellular ATP-levels are perturbed in Δ*relA* and Δ*relA/spoT* cells. Measurements of intracellular ATP levels in MG1655 wt (green), Δ*relA* (orange) and Δ*relA/spoT* (red) during different growth phases using the ratiometric fluorescent ATP-reporter QUEEN-7 µ. >100 cells were measured per sample. The mean is and SD are indicated by the black line and error bars, respectively.

During active growth, adenylate kinase converts AMP to ADP, the latter of which is fed into the ATP biosynthesis pathway. Considering the indications that the SR responded to disturbances in this pathway, we set out to investigate if ATP homeostasis was intrinsically offset in (p)ppGpp-deficient cells. Employing the intracellular ratiometric fluorescent ATP-sensor QUEEN-7 µ^29^, we measured the ATP levels in wt, Δ*relA* and Δ*relA/spoT* during different growth phases (Fig. 7). Samples were taken from LB cultures in mid log-, early stationary- and late stationary phase (>20 h), and the emission levels of the fluorescent reporter was measured in >100 individual cells in three independent experiments.

In agreement with previously reported findings, ATP levels remained constant in mid log- and early stationary phase in wt^30^, whereas they dropped significantly in late stationary phase^31,32^ (Fig. 7). Isogenic Δ*relA* mutants exhibited largely the same phenotype as wt during the two initial time points; however, they were unable to reduce the ATP concentrations in late stationary phase, implying that RelA has a key role in this process. In contrast, the Δ*relA/spoT* strain displayed severely altered ATP levels in all growth phases, being below wt concentrations during mid log- and early stationary phase, but above them in late stationary phase, appearing unable to regulate ATP turnover. In summary, our results strongly suggest that (p)ppGpp is crucial for a normal cellular energy homeostasis in *E. coli* MG1655.

## Discussion

To ensure that the building blocks for all the simultaneously biosynthesized cellular components are concurrently present, cell growth needs to be highly coordinated. The SR is a central stress pathway in *E. coli* that globally regulates macromolecular synthesis^2,5^. During amino acid starvation, the best studied model for SR activation, the effector alarmone (p)ppGpp is synthesized and transiently halts macromolecular biosynthesis to greatly decelerate cellular growth rates in order to allow the amino acid pools to recover to growth-permissive levels, ensuring that nutrients are present in sufficient quantities for coordinated growth. In a similar manner, albeit mechanistically less understood, the SR is activated upon starvation for carbon sources, fatty acids and phosphate^2,33^. Collectively, these findings implicate the SR as having a central role in the coordination of cell growth.

Whereas activation of the SR has been extensively studied in response to exogenous stresses in *E. coli*, its role in responding to intracellular imbalances is less investigated. Such studies may have in part been limited by the lack of efficient tools to broadly study depletion of essential gene products that are often involved in central cellular processes. Here, we modified the CRISPRi transcriptional repression method to acquire an efficient and reliable tool for studying essential genes in *E. coli* MG1655 (Supplementary Fig. S1). Applying the method, we found that (p)ppGpp accumulation does not generally occur during stresses that affect cell viability, but rather transpires during specific intracellular cues during active growth, namely disturbances in adenosine ribonucleotide metabolism and outer membrane biogenesis (Figs 2 and 3**)**. There were also indications that the SR might be involved in the response to phospholipid biosynthesis defects (Fig. 2A,B). Nucleotide- and LPS biosynthesis have previously been reported to be regulated by (p)ppGpp during starvation^5^, but, to the best of our knowledge, this is the first study that shows that aberrations in these pathways also induce the SR in *E. coli*, indicating a feedback mechanism.

Lipopolysaccharides are essential components of the *E. coli* cell envelope, constituting the major structural building blocks of the outer leaflet of the outer membrane^34^. Monomers of LPS are synthesized in the cytoplasm, flipped to the periplasm by the MsbA translocator, and transported to the outer membrane for insertion by the Lpt-system^35^. LpxA catalyzes the first reaction in the biosynthetic pathway of lipid A, the hydrophobic anchor-domain of LPS^34^. Our data shows that if the synthesis of lipid A is disturbed via repression of *lpxA*, (p)ppGpp is synthesized (Figs 2 and 3**)**. Alarmone synthesis was also observed during repression of *lptA*, whose encoded product is a periplasmic component of the Lpt-system that ferries LPS monomers to the outer membrane for insertion^35^ (Figs 2 and 3**)**. During the transcriptional repression of both these genes, cell division ceases rapidly (Fig. 4), indicating the existence of a designated stress response that halts growth when encountering imbalances in outer membrane biogenesis. In comparison, cells experiencing repression of *murC*, encoding an enzyme involved in cell wall biosynthesis, could not detect the imbalance and proceeded with untimely division, resulting in rapid lysis (Supplementary Fig. S2L).

We could also show that (p)ppGpp^0^ cells could not aptly respond to the disturbances caused by *lptA* and *lpxA* repression, instead growing in an uncoordinated manner until lysis occurred (Fig. 6). Interestingly, the (p)ppGpp effect was indicated to be RelA-independent in these conditions, implicating the involvement of SpoT in the process. Moreover, genes of the *lpx*-operon have previously been shown to be some of the relatively few genetic elements differentially regulated during amino acid starvation in Δ*relA/spoT* compared to Δ*relA* isogenic strains^5^, further indicating a connection to SpoT. It remains to be mechanistically elucidated if signals for perturbations in these pathways are relayed via SpoT.

Adenylate kinase is the only enzyme that can recycle cellular AMP to the ATP synthase-substrate ADP, and has been postulated to be a central growth rate controlling protein^36^. Interestingly, it has been shown that when a thermo-sensitive adenylate kinase-producing *E. coli* is cultured at non-permissive temperatures, macromolecular biosynthesis decreases rapidly^36^. Our data suggests that repression of *adk* induces (p)ppGpp synthesis in a RelA-dependent manner and that this pathway is required to potently inhibit growth (Figs 2–5).

The cellular levels of AMP correspond to approx. 4% of the ATP concentration in mid log phase^30^. At the same time, AMP is known to regulate many enzymes^37–41^, of which the best studied example is fructose-1,6-bisphosphatase involved in gluconeogenesis^42^. Moreover, one study has previously reported that AMP inhibits the arginine-tRNA ligase ArgRS *in vitro*^43^, meaning that (p)ppGpp accumulation in response to disturbances in ATP metabolism may be “routed” via the classically described stringent response activation pathways. Considering these facts, it is possible that repression of *adk* results in a significant increase in AMP concentration that leads to the inhibition of anabolic processes and the induction of the SR, in this way coordinating macromolecular synthesis with adenosine ribonucleotide availability during normal growth. It remains to be experimentally determined if this is the case.

Should (p)ppGpp be involved in coordinating adenosine ribonucleotide synthesis with macromolecular production, (p)ppGpp^0^ cells unable to harmonize these cellular pathways could be expected to exhibit aberrations in ATP levels. Our data strongly suggests that this is indeed the case (Fig. 7). *In vivo* measurements of intracellular ATP concentrations indicate that RelA is required to drop ATP levels in late stationary phase, whereas SpoT potentially plays a larger role in synchronizing cellular processes during normal growth, as the isogenic Δ*relA/spoT* had considerably lower ATP-levels compared to wt and Δ*relA* in mid log phase, but both mutants were incapable of reducing ATP levels in late stationary phase. The lower ATP levels associated with the Δ*relA/spoT* could potentially account for the previously observed reduced growth rate of the strain. As the complete lack of the alarmone potentially causes an overuse of ATP in mid log phase, our findings thus suggest that (p)ppGpp plays a role in coordinating resource usage during active growth.

In summary, our results elucidate novel roles for the SR in *E. coli* physiology as a responder to intracellular metabolic cues involving ADP metabolism and LPS biosynthesis, safeguarding against uncoordinated growth with regard to these processes. The molecular pathways that lead to the activation of the SR during these imbalances will be highly interesting to investigate in the future.

## Material and Methods

### Bacterial strains and growth conditions

All strains used in this study were derivatives of *E. coli* K-12 strain MG1655 and are listed in Supplementary Table S2. The origin of the MG1655 Δ*relA/spoT* strain used was *E. coli* PDC47^44^, in which the selection marker was changed from chloramphenicol- to zeocin resistance (to render the strain compatible with the CRISPRi system) via λ-red recombineering^45^. MG1655 Δ*relA* was constructed via the insertion of a kanamycin resistance gene from the Keio collection strain JW2755 in the designated *relA* locus using P1 transduction as previously described^46^. Strain MG1655 Δ*relA::kan* was transformed with pCP20 and incubated at 42 °C overnight to remove the kanamycin resistance cassette flanked by FRT sites. All genetic constructs were verified using standard polymerase chain reaction (PCR) and DNA sequencing. Bacteria were routinely cultured at 37 °C in lysogeny broth (LB) or M9 minimal medium supplemented with 2% glucose, 10 µg/ml thiamine and 0.1 µg/ml amino acids (excluding Phe, Tyr, and Trp to limit background fluorescence). Chloramphenicol (25 µg/ml), ampicillin (50 µg/ml) and zeocin (60 µg/ml) were used for selection when applicable. For induction of CRISPRi, anhydrotetracycline (aTc) was used (final concentration 1 µM).

### CRISPRi modification and pgRNA constructs

The plasmid pdCas9-ssrA was created by PCR amplifying the 5′ end of *dcas9* using the dcasUP (GAAATTCGGACAAGCTTATTGCTCGT) and dcasSSRA (TCCTTACTCGAGTTATCATTAAGCTGCTAAAGCGTAGTTTTCGTCGTTTGCTGCGTCACCTCCTAGCTGACTCAAATC) primers, and replacing the *XhoI-BamHI* fragment in pdCas9-Bacteria^12^ with the amplified fragment. All pgRNA were constructed by insertion of gene-specific N_20_ targeting regions (listed in Supplementary Table S1) between the *SpeI* and *HindIII* restriction sites via standard cloning techniques using customized DNA oligonucleotides.

### Time-lapse fluorescence microscopy

Phase contrast and fluorescence microscopy was performed using Nikon Eclipse Ti inverted microscope with a motorized automatic stage. In short, cells were taken in steady state growth and inoculated on 1% agarose-pads with M9- (CRISPRi screen) or LB medium ((p)ppGpp^0^ cell imaging), supplemented with the indicated inducers and selection markers when applicable. The agarose-pads were transferred to cover slips and sealed via a custom setup to allow imaging over prolonged periods of time. The samples were then transferred to the microscope enclosure and allowed to adjust to 37 °C for 30 min. Phase contrast and fluorescence images were recorded every 10–20 minutes for up to 17 hours at 37 °C in temperature-controlled settings. The excitation and emission wavelengths used were 580/615 (mCherry, 500 ms), and 405/520 and 490/520 (QUEEN-7 µ ATP-sensor, 500 ms and 1 s, respectively). The images were analyzed, fluorescence measured and movies created using the Nikon NIS Elements software.

### *In vivo* (p)ppGpp measurements

Cellular (p)ppGpp levels were determined as previously described^47^. In brief, strains were brought to steady state in and subsequently diluted to OD_600_ = 0.01 in LB. Radioactive H_3_^32^PO_4_ (200 µCi ml^−1^) was added at this time point and the cultures allowed to grow for approx. 3 generations at 37 °C with shaking. *dcas9* was subsequently induced and samples were taken at the stated time points. The samples were processed and thin-layered chromatography (TLC) was performed as previously described^47^.

### CRISPRi survival kinetics assay

Overnight cultures of CRISPRi strains were subcultured in LB and brought to steady state growth. Subsequently, the cultures were diluted back to OD_600_ = 0.01 and *dcas9* was induced. The cultures were incubated at 37 °C with shaking and samples were taken with 30 min intervals for two hours and plated out for viable count. Samples were also plated on aTc-plates for control to monitor potential repressor mutants that escaped CRISPRi. The experiment was performed in three independent replicates and the data obtained plotted.

### Nucleoid staining for microscopy

Cellular nucleoids were stained with 4′, 6-diamidino-2-phenylindole (DAPI) as previously described^48^. In short, cells were taken in balanced growth and inoculated in liquid supplemented M9 minimal medium with *dcas9*-inducing aTc at 37 °C without shaking. At 3 h and 10 h post-induction, samples were taken and stained with DAPI (500 nM final concentration) for 10 min at room temperature. Samples were imaged with phase contrast and fluorescence microscopy as described^48^.

## Supplementary information

Supplementary information
