# Supplementary material for: (p)ppGpp-mediated stress response induced by defects in outer membrane biogenesis and ATP production promotes survival in Escherichia coli
Source: Sci Rep. 2019 Feb 27;9:2934. doi: 10.1038/s41598-019-39371-3 (PMC6393671; doi:10.1038/s41598-019-39371-3)
Supplement: Supplementary file 1 — Supplementary information [file 41598_2019_39371_MOESM1_ESM.pdf]

Supplementary information

(p)ppGpp-mediated stress response induced by defects in outer membrane biogenesis and ATP production promotes survival in *Escherichia coli*

Mohammad Roghanian[1], Szabolcs Semsey[1], Anders Løbner-Olesen[1] & Farshid Jalalvand[1]*

[1]Centre for Bacterial Stress Response and Persistence, Department of Biology, University of Copenhagen, Ole Maaløes Vej 5, DK-2200 Copenhagen, Denmark

*Corresponding author: Farshid Jalalvand, PhD, farshid.jalalvand@bio.ku.dk

A

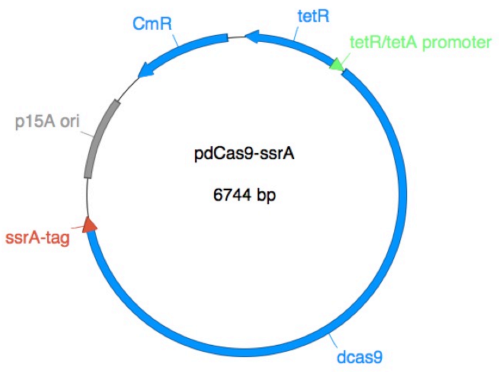

B

AANDENYALAA

C

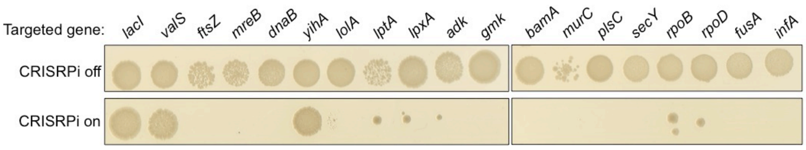

Figure S1. Fusion of ssrA degradation-tag to *dcas9*. (**A**) Schematic plasmid map of pdCas9-ssrA. (**B**) The amino acid sequence of the ssrA degradation-tag fused to dCas9. (**C**) Viability of *E. coli* MG1655 containing pdCas9-ssrA and sgRNA against the indicated genes. Overnight cultures were diluted 1000x and 3 µl spotted on *dcas9-ssrA* non-inducing (CRISPRi off) and inducing (CRISPRi on) solid LB media.

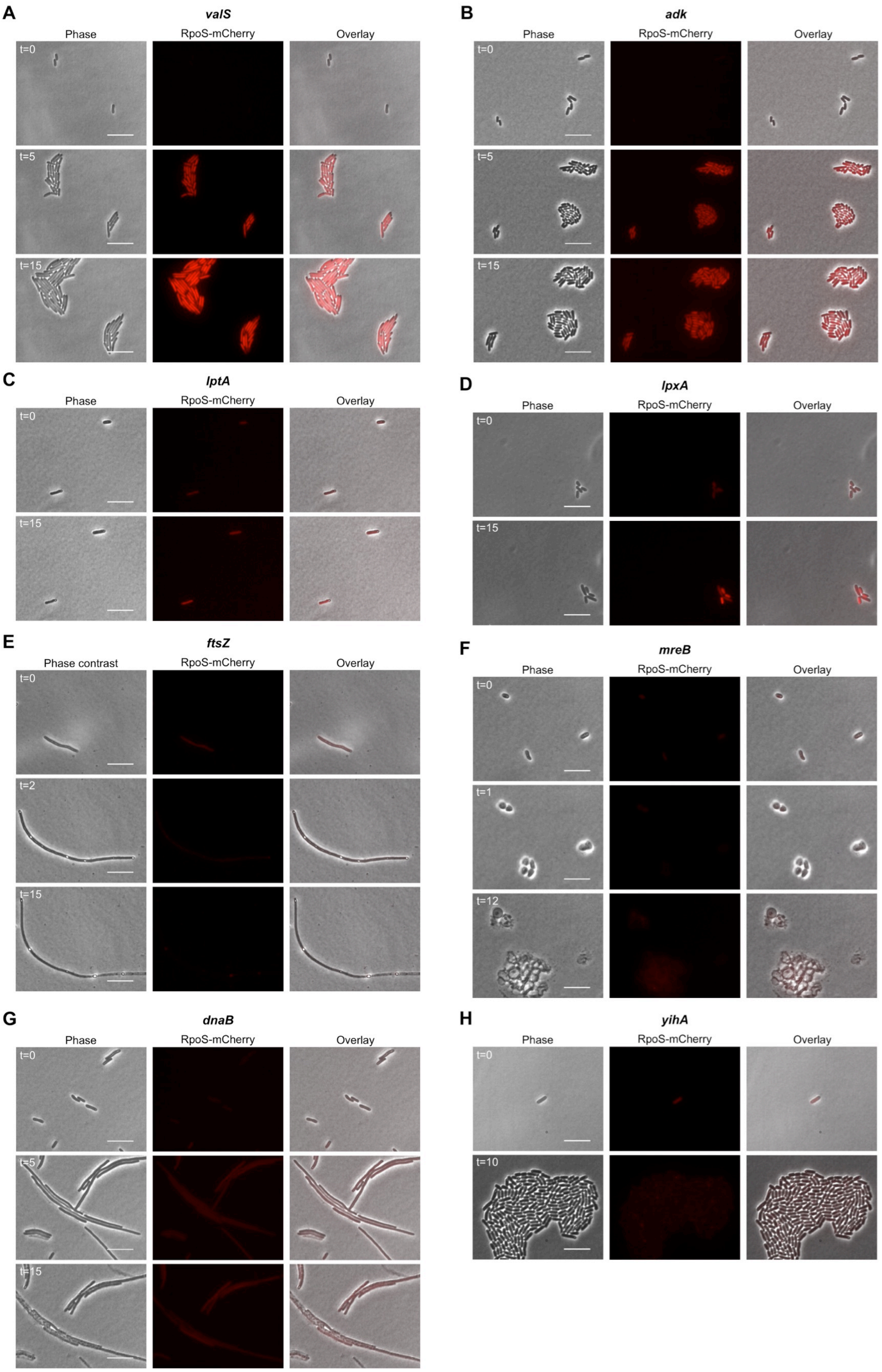
A
valS
Phase
RpoS-mCherry
Overlay
t=0
t=5
t=15
B
adk
Phase
RpoS-mCherry
Overlay
t=0
t=5
t=15
C
lptA
Phase
RpoS-mCherry
Overlay
t=0
t=15
D
lpxA
Phase
RpoS-mCherry
Overlay
t=0
t=15
E
ftsZ
Phase contrast
RpoS-mCherry
Overlay
t=0
t=2
t=15
F
mreB
Phase
RpoS-mCherry
Overlay
t=0
t=1
t=12
G
dnaB
Phase
RpoS-mCherry
Overlay
t=0
t=5
t=15
H
yihA
Phase
RpoS-mCherry
Overlay
t=0
t=10

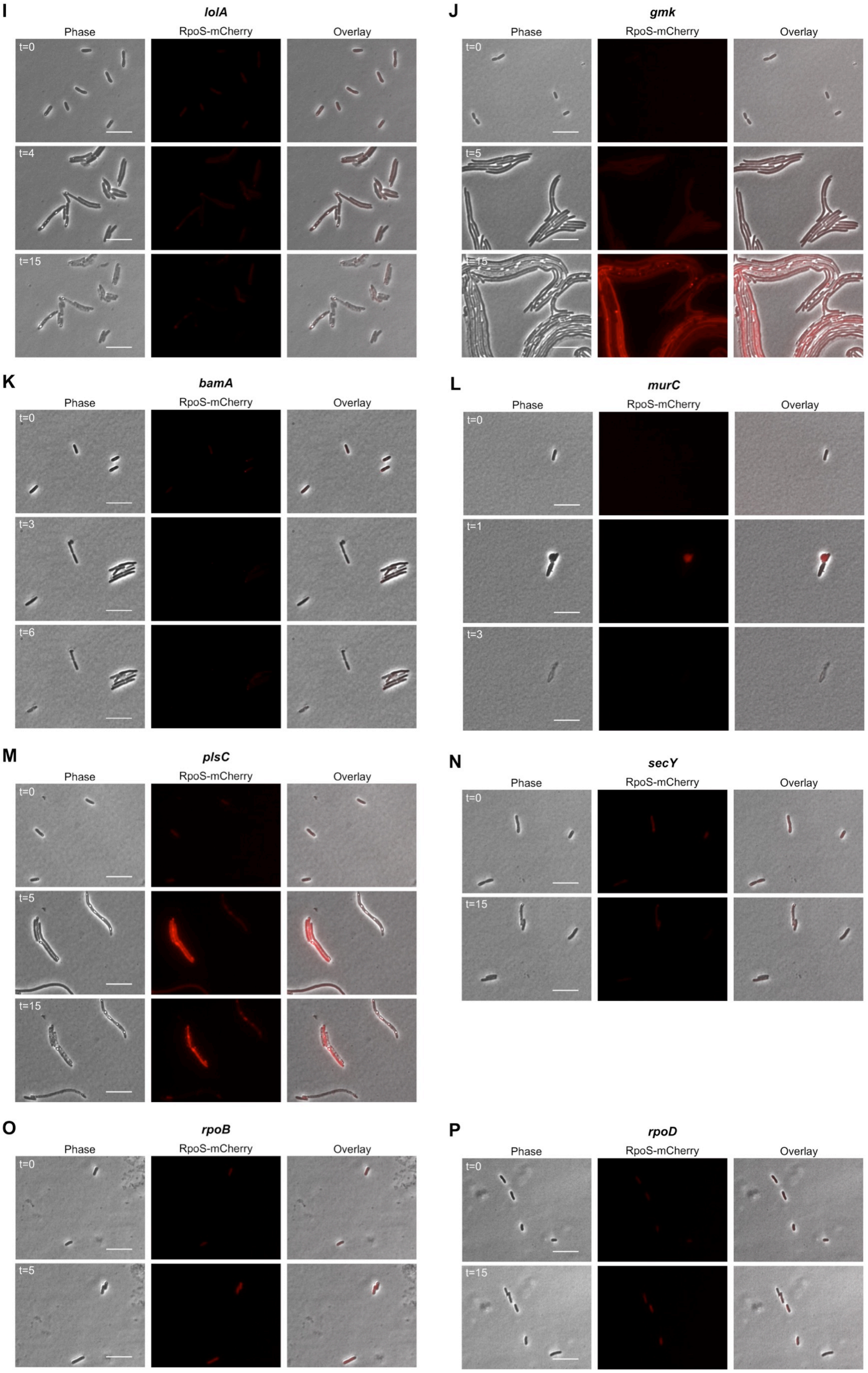
I
lolA
Phase
RpoS-mCherry
Overlay
t=0
t=4
t=15
J
gmk
Phase
RpoS-mCherry
Overlay
t=0
t=5
t=15
K
bamA
Phase
RpoS-mCherry
Overlay
t=0
t=3
t=6
L
murC
Phase
RpoS-mCherry
Overlay
t=0
t=1
t=3
M
plsC
Phase
RpoS-mCherry
Overlay
t=0
t=5
t=15
N
secY
Phase
RpoS-mCherry
Overlay
t=0
t=15
O
rpoB
Phase
RpoS-mCherry
Overlay
t=0
t=5
P
rpoD
Phase
RpoS-mCherry
Overlay
t=0
t=15

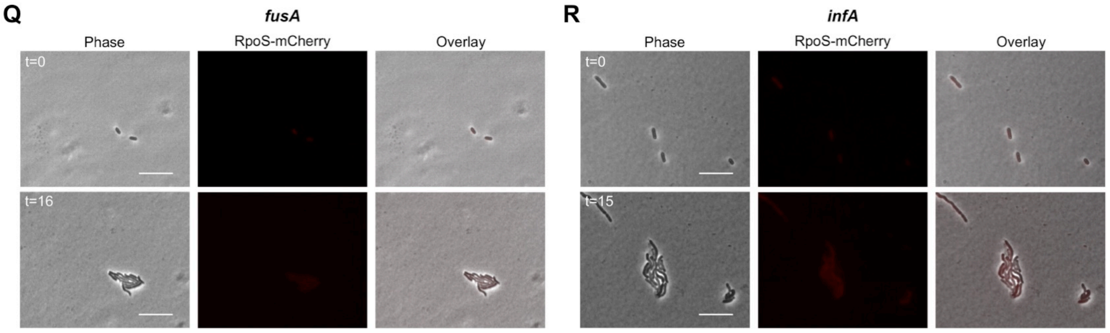

Figure S2. Time-lapse fluorescence microscopy images during repression of the surveyed genes. CRISPRi-mediated shut down of expression of the indicated genes in a MG1655-derivate strain harboring the intracellular (p)ppGpp-reporter *rpoS-mCherry.* Phase contrast (left panels), the (p)ppGpp-reporter RpoS-mCherry fluorescence (middle panels) and the merged overlay images (right panels) capturing cells throughout the duration of the experiment. Scale bars = 10 μm. The repressed genes are indicated in bold text: (**A**) *valS* (**B**) *adk* (**C**) *lptA* (**D**) *lpxA* (**E**) *ftsZ* (**F**) *mreB* (**G**) *dnaB* (**H**) *yihA* (**I**) *lolA* (**J**) *gmk* (**K**) *bamA* (**L**) *murC* (**M**) *plsC* (**N**) *secY* (**O**) *rpoB* (**P**) *rpoD* (**Q**) *fusA*, and (**R**) *infA*.

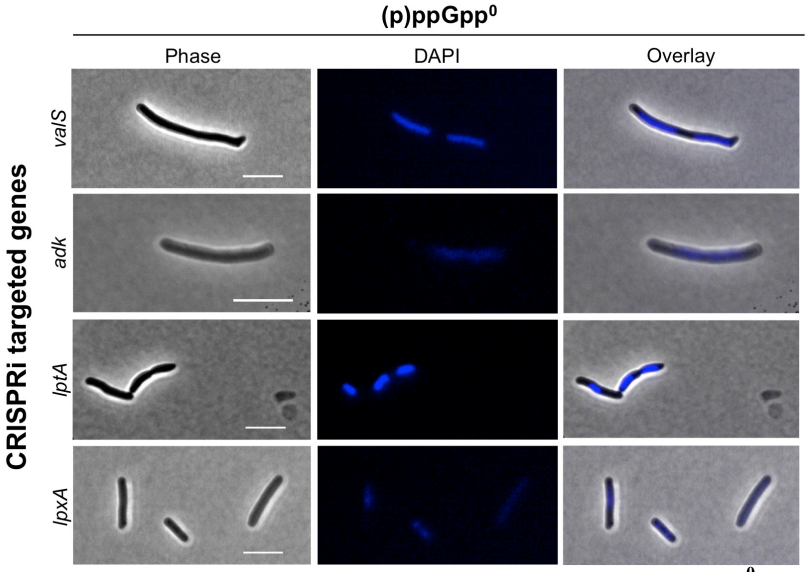

Figure S3. The nucleoid localizes differently in (p)ppGpp^0 cells during repression of *valS*, *adk*, *lptA* and *lpxA*. Fluorescence microscopy imaging of DAPI-stained Δ*relA/spoT* cells 3h post CRISPRi-mediated transcriptional repression of the indicated genes. Phase contrast (left panels), DAPI fluorescence (middle panels) and their merged overlay (right panels) are shown. Scale bars = 10 μm.

Table S1. The selected cellular processes targeted in this study. The involved genes, corresponding functions, locus tags and the gRNA targeting regions are listed.

Cellular process	Gene	Function	Locus tag	sgRNA N_{20} region	Ref
tRNA-charging (positive control)	*valS*	Valine-tRNA ligase	b4258	gcggctgttcgatatcttgt	1
Cell division	*ftsZ*	Septal ring protein	b0095	ctttcagcagtttgtcgttc	2
Cytoskeleton	*mreB*	Structural cytoskeletal protein	b3251	ttgtccttttacataaatga	3
DNA replication	*dnaB*	Replicative DNA helicase	b4052	tcagcctgctgtttgttgaa	4
Essential GTPases	*yihA*	Function not fully established	b3865	gataagctgggtgcgccctg	5
Lipoprotein insertion in outer membrane	*lolA*	Outer membrane lipoprotein carrier protein	b0891	ccgtagcttgctcaacgaac	6
LPS biosynthesis/transport	*lptA*	Periplasmic subunit of the Lpt LPS-transport system	b3200	attaattttgatggtgccct	7
LPS biosynthesis/transport	*lpxA*	UDP-N-acetylglucosamine acyltransferase, lipid A biosynthesis pathway	b0181	accctcaccaatttcgacat	8
Nucleotide metabolism	*adk*	Adenylate kinase	b0474	ctgcctgcggaatggtacgc	9
Nucleotide metabolism	*gmk*	Guanylate kinase	b3648	ggatttacccgcgccactgg	10
Outer membrane protein assembly	*bamA*	Essential BAM system component for assembly of proteins in the outer membrane	b0177	gcgcaccggcatactgagga	11
Peptidoglycan synthesis	*murC*	UDP-*N*-acetylmuramate—L-alanine ligase, lipid II biosynthesis pathway	b0091	tggcatcacgtacgttttcc	12
Phospholipid biosynthesis	*plsC*	1-acylglycerol-3-phosphate *O*-acyltransferase, phospholipid biosynthesis pathway	b3018	ccacatgtttcgggttacgc	13
Secretion system	*secY*	Sec translocon subunit SecY	b3300	gtacagcggcatcaatacca	14
Transcription	*rpoB*	RNA polymerase subunit β	b3987	taccgctgtagctctgaatc	15
Transcription	*rpoD*	Sigma 70, exponential growth sigma factor	b3067	gaagtttcagctgtgactgc	16
Translation	*fusA*	Translation elongation factor G	b3340	atgttacggtagcgtgcgat	17
Translation	*infA*	Translation initiation factor IF-1	b0884	attaggcaacgtttcaagaa	18
Lactose metabolism (negative control)	*lacI*	Transcriptional repressor of the *lac*-operon	b0345	gctggcctggttcaccacgc	19

Table S2. Strains and plasmids used in this study.

Strain/plasmid	Genotype	Source
Strains		
E. coli K-12 MG1655	Wild-type	Laboratory collection
::*rpoS-mCherry*	MG1655 *rpoS::mcherry-frt-aphA-frt*	[20]
Δ*relA*	Δ*relA::*FRT	This study
Δ*relA/spoT*	Δ*relA*::FRT/*spoT::zeoR*	This study
Plasmids		
pdCas9-ssrA	P_{tet}::*dcas9-ssrA*	This study
pgRNA-lacI	See Table S1 for N_{20} matching sequences	This study
pgRNA-valS	See Table S1 for N_{20} matching sequence	This study
pgRNA-adk	See Table S1 for N_{20} matching sequence	This study
pgRNA-lptA	See Table S1 for N_{20} matching sequence	This study
pgRNA-lpxA	See Table S1 for N_{20} matching sequence	This study
pgRNA-ftsZ	See Table S1 for N_{20} matching sequence	This study
pgRNA-mreB	See Table S1 for N_{20} matching sequence	This study
pgRNA-dnaB	See Table S1 for N_{20} matching sequence	This study
pgRNA-yihA	See Table S1 for N_{20} matching sequence	This study
pgRNA-lolA	See Table S1 for N_{20} matching sequence	This study
pgRNA-gmk	See Table S1 for N_{20} matching sequence	This study
pgRNA-bamA	See Table S1 for N_{20} matching sequence	This study
pgRNA-murC	See Table S1 for N_{20} matching sequence	This study
pgRNA-plsC	See Table S1 for N_{20} matching sequence	This study
pgRNA-secY	See Table S1 for N_{20} matching sequence	This study
pgRNA-rpoB	See Table S1 for N_{20} matching sequence	This study
pgRNA-rpoD	See Table S1 for N_{20} matching sequence	This study
pgRNA-fusA	See Table S1 for N_{20} matching sequence	This study
pgRNA-infA	See Table S1 for N_{20} matching sequence	This study

Supplementary References

1 Kaplan, S., Atherly, A. G. & Barrett, A. Synthesis of stable RNA in stringent Escherichia coli cells in the absence of charged transfer RNA. *Proceedings of the National Academy of Sciences of the United States of America* **70**, 689-692 (1973).

2 Romberg, L. & Levin, P. A. Assembly dynamics of the bacterial cell division protein FTSZ: poised at the edge of stability. *Annual review of microbiology* **57**, 125-154, doi:10.1146/annurev.micro.57.012903.074300 (2003).

3 Wachi, M. *et al.* Mutant isolation and molecular cloning of mre genes, which determine cell shape, sensitivity to mecillinam, and amount of penicillin-binding proteins in Escherichia coli. *Journal of bacteriology* **169**, 4935-4940 (1987).

4 Kaguni, J. M. *et al.* Initiation of replication of the Escherichia coli chromosomal origin reconstituted with purified enzymes. *Basic Life Sci* **30**, 141-150 (1985).

5 Dassain, M., Leroy, A., Colosetti, L., Carole, S. & Bouche, J. P. A new essential gene of the 'minimal genome' affecting cell division. *Biochimie* **81**, 889-895 (1999).

6 Tajima, T., Yokota, N., Matsuyama, S. & Tokuda, H. Genetic analyses of the in vivo function of LolA, a periplasmic chaperone involved in the outer membrane localization of Escherichia coli lipoproteins. *FEBS Lett* **439**, 51-54 (1998).

7 May, J. M., Sherman, D. J., Simpson, B. W., Ruiz, N. & Kahne, D. Lipopolysaccharide transport to the cell surface: periplasmic transport and assembly into the outer membrane. *Philos Trans R Soc Lond B Biol Sci* **370**, doi:10.1098/rstb.2015.0027 (2015).

8 Galloway, S. M. & Raetz, C. R. A mutant of Escherichia coli defective in the first step of endotoxin biosynthesis. *The Journal of biological chemistry* **265**, 6394-6402 (1990).

9 Glaser, M., Nulty, W. & Vagelos, P. R. Role of adenylate kinase in the regulation of macromolecular biosynthesis in a putative mutant of Escherichia coli defective in membrane phospholipid biosynthesis. *Journal of bacteriology* **123**, 128-136 (1975).

10 Hible, G. *et al.* Calorimetric and crystallographic analysis of the oligomeric structure of Escherichia coli GMP kinase. *Journal of molecular biology* **352**, 1044-1059, doi:10.1016/j.jmb.2005.07.042 (2005).

11 Wu, S. *et al.* Biofilm-specific extracellular matrix proteins of nontypeable Haemophilus influenzae. *Pathogens and disease*, doi:10.1111/2049-632X.12195 (2014).

12 El Zoeiby, A., Sanschagrin, F. & Levesque, R. C. Structure and function of the Mur enzymes: development of novel inhibitors. *Molecular microbiology* **47**, 1-12 (2003).

13 Parsons, J. B. & Rock, C. O. Bacterial lipids: metabolism and membrane homeostasis. *Prog Lipid Res* **52**, 249-276, doi:10.1016/j.plipres.2013.02.002 (2013).

14 Park, E. *et al.* Structure of the SecY channel during initiation of protein translocation. *Nature* **506**, 102-106, doi:10.1038/nature12720 (2014).

15 Wang, Y. *et al.* Determinants for Escherichia coli RNA polymerase assembly within the beta subunit. *Journal of molecular biology* **270**, 648-662, doi:10.1006/jmbi.1997.1139 (1997).

16 Jishage, M., Iwata, A., Ueda, S. & Ishihama, A. Regulation of RNA polymerase sigma subunit synthesis in Escherichia coli: intracellular levels of four species of sigma subunit under various growth conditions. *Journal of bacteriology* **178**, 5447-5451 (1996).

17 Wintermeyer, W., Savelsbergh, A., Semenkov, Y. P., Katunin, V. I. & Rodnina, M. V. Mechanism of elongation factor G function in tRNA translocation on the ribosome. *Cold Spring Harb Symp Quant Biol* **66**, 449-458 (2001).

18 Cummings, H. S. & Hershey, J. W. Translation initiation factor IF1 is essential for cell viability in Escherichia coli. *Journal of bacteriology* **176**, 198-205 (1994).

19 Lewis, M. The lac repressor. *C R Biol* **328**, 521-548, doi:10.1016/j.crvi.2005.04.004 (2005).

20 Maisonneuve, E., Castro-Camargo, M. & Gerdes, K. (p)ppGpp controls bacterial persistence by stochastic induction of toxin-antitoxin activity. *Cell* **154**, 1140-1150, doi:10.1016/j.cell.2013.07.048 (2013).
